# Parkinson's disease-associated psychosis in a Brazilian sample: prevalence and clinical profile

**DOI:** 10.1055/s-0046-1829035

**Published:** 2026-10-06

**Authors:** Henrique Nascimento Dourado, Isabelle Jacqueline Weber Oliveira, Camila Caroso Lobo, Gustavo Cavalcante Procópio, Ana Emanuela Coelho Pessoa Lima, Guilherme Teixeira Valença

**Affiliations:** 1 Centro de Neurociências, Escola Bahiana de Medicina e Saúde Pública, Salvador BA, Brazil.; 2 Hospital Geral Roberto Santos, Ambulatório de Distúrbios do Movimento e Doença de Parkinson, Salvador BA, Brazil.

**Keywords:** Parkinson Disease, Psychotic Disorders, Neuropsychiatry, Prevalence

## Abstract

**Background:**

Psychosis is a frequent and disabling non-motor complication of Parkinson's disease (PD). Reported prevalence varies widely, and data in Brazilian cohorts remain scarce.

**Objective:**

To estimate the prevalence of PD-associated psychosis (PDP) in a Brazilian cohort and to describe its sociodemographic and clinical profile.

**Methods:**

We conducted a cross-sectional study in a Brazilian movement disorders outpatient clinic between May and December of 2022. Adult patients with idiopathic PD defined by the United Kingdom's Parkinson's Disease Society Brain Bank criteria were consecutively evaluated. Sociodemographic and clinical variables were collected. Psychosis was assessed using the diagnosis criteria proposed by the National Institute of Neurological Disorders and Stroke and the National Institute of Mental Health (NINDS/NIMH) and graded with the Movement Disorders Society's Unified PD Rating Scale (MDS-UPDRS). Cognitive screening was performed using the Mini-Mental State Examination (MMSE), and disease severity was assessed with the modified Hoehn & Yahr scale (mH&Y). Neuropsychiatric symptoms were evaluated with the Neuropsychiatric Inventory Questionnaire (NPI-Q). Descriptive analyses and exploratory univariate comparisons were performed.

**Results:**

In total, 75 patients were included. The median age was 68 years (IQR: 59.5–74.5), and the mean age at PD onset was 56.0 ±  9.8 years. Median disease duration was 10 years (IQR: 6–14.5). Most patients were male (54.7%) and self-identified as Mixed-race (48.6%) or Black (31.1%). According to the mH&Y scale, 82.7% had mild-to-moderate disease. Furthermore, PDP was diagnosed in 18 patients (24%) according to NINDS/NIMH criteria. Minor phenomena and hallucinations were the most frequent psychotic manifestations, each occurring in 72.2% of PDP cases.

**Conclusion:**

In this Brazilian cohort, PDP had a prevalence of 24%. These findings reinforce that psychosis is a frequent non-motor manifestation of PD and highlight the importance of systematic clinical screening in routine care.

## INTRODUCTION

Parkinson's disease (PD) psychosis (PDP) negatively impacts patients and caregiver's quality of life, contributing to disability and posing an increased risk of patient admission to a nursing home. 1 Characteristically, it initially presents as ‘minor hallucinatory phenomena’ (illusions, false sense of presence, and passage hallucinations), which may progress to formed hallucinations with preserved insight, subsequently evolving into loss of insight and other types of hallucinations, ultimately presenting with delusions and illusions. 2 Furthermore, PDP carries a poor prognosis and has been associated with the development of dementia, greater disability, mortality, caregiver burden, and, consequently, a higher likelihood of nursing home admission. 1 3 4 5 6

Although its pathophysiology is not fully elucidated, the current consensus is that PDP is intrinsic to the disease, being potentially influenced by dopaminergic replacement therapy, and its onset can be triggered or ‘accelerated’ by it. 7 8 Thus, it is accepted that the development of psychosis is a combination of exogenous factors, such as treatment, and endogenous factors inherent to the disease and the patient. 1 While there is no clarity regarding its pathophysiology, its development has been consistently linked to cognitive impairment, more severe PD, longer disease duration, advanced age, and sleep disorders. 1 Notably, hallucinations have also been associated with the postural instability and gait difficulty (PIGD) motor subtype and identified as a predictor of future freezing of gait. 9 Other associations found include autonomic dysfunction, female sex, as well as depression, anxiety, and visual impairment. 2 10

Estimates of PDP prevalence vary widely across studies due to methodological differences between studies, a lack of consensus in PDP definition, varying samples, assessment timeframes and locations, and the use of medications that can induce psychosis as side effects. 3 However, it is estimated to range from 13 to 60% in cross-sectional studies, depending on the population and diagnostic criteria used, while cumulative lifetime prevalence may reach 47 to 60%. 1 Its incidence has been estimated to be between 4.3 and 8.0 per 100 person-years in longitudinal cohorts. 6 11

In 2007, the National Institute of Neurological Disorders and Stroke (NINDS) and the National Institute of Mental Health (NIMH) proposed broader diagnostic criteria for PDP, replacing the earlier DSM-based definitions. 4 However, only fair agreement has been observed between NINDS/NIMH and Diagnostic and Statistical Manual of Mental Disorders (DSM) criteria, raising concerns about their validity. 12 More recently, the Movement Disorder Society (MDS) has advanced efforts toward standardization, presenting the prototype of the Parkinson's Disease Psychosis Rating Scale (MDS-PDPRS), in 2024, a clinician-reported outcome designed to quantify PDP severity and functional impact. 13

Despite extensive international literature, data on PDP in Brazilian patients remain scarce. Therefore, the present study aimed to determine the prevalence of PDP and to describe the sociodemographic and clinical profile of a Brazilian Parkinson's disease cohort. As a secondary objective, we conducted an exploratory analysis of clinical and treatment-related factors potentially associated with PDP.

## METHODS

### Study design and setting

This was a cross-sectional study conducted at the Movement Disorders Outpatient Clinic of Hospital Geral Roberto Santos, a tertiary referral center in Salvador, Bahia, Brazil. The study was performed between May and December 2022. The primary objective was to estimate the prevalence of PDP in a Brazilian tertiary cohort and to describe its sociodemographic and clinical profile. As a secondary exploratory assessment, we investigated potential clinical and treatment-related factors associated with the presence of PDP. The reporting of this study follows the Strengthening the Reporting of Observational Studies in Epidemiology (STROBE) statement's recommendations for cross-sectional studies. 14

### Participants

Eligible participants were aged 18-years or older, of any sex, with a diagnosis of idiopathic PD according to the United Kingdom Parkinson's Disease Society Brain Bank (UKBB) criteria. 15 Diagnosis was confirmed by a neurologist specialized in movement disorders. Patients were excluded if they presented with aphasia, a history of multiple head traumas, secondary parkinsonism, other movement disorders, or neuropsychiatric comorbidities that could confound the diagnosis of psychotic symptoms (e.g., bipolar disorder). A total of 75 patients were consecutively enrolled as a convenience sample.

### Variables and measurements

Sociodemographic and clinical data were obtained through a standardized structured questionnaire and review of medical records. The presence of PDP was defined according to the NINDS/NIMH criteria, which require at least one psychotic symptom (illusions, false sense of presence, hallucinations, or delusions) occurring recurrently or continuously for at least 1 month, after exclusion of other causes of psychosis. 4

Notably, although passage hallucinations are considered a minor hallucinatory phenomenon, they are not included in the NINDS/NIMH criteria. Therefore, they were not considered for the diagnosis of PDP. The use of these criteria is currently recommended by the MDS to standardize PDP assessment. 16 Psychosis severity was graded using the MDS-UPDRS Part I, item 1.2. 17

Additional neuropsychiatric symptoms and caregiver burden were evaluated using the Neuropsychiatric Inventory Questionnaire (NPI-Q). 18 19 Disease severity was assessed using the modified Hoehn & Yahr (mH&Y) scale, with stages 1 to 3 classified as mild-to-moderate disease and stages 4 to 5 as severe disease. 20 The Mini-Mental State Examination (MMSE) was used for cognitive screening, as it remains a widely accessible and validated tool in the Brazilian clinical context. 21 22

Levodopa equivalent daily dose (LEDD) was calculated according to standardized conversion factors. 23 The following multipliers were applied: immediate-release levodopa (×1.0), controlled-release levodopa (×0.75), and dual-release levodopa (×0.85). The use of entacapone, a catechol-O-methyltransferase inhibitor (COMTi), increased the corresponding levodopa dose by 0.33. Both rasagiline, a monoamine oxidase B inhibitor (MAO-Bi), and pramipexole, a dopamine agonist, were converted using a multiplier of ×100, whereas amantadine was considered equivalent to its daily dose (×1.0). Finally, LEDD was coded as zero for patients not receiving any antiparkinsonian medication.

### Bias control

To reduce bias, all assessments were performed by the same trained researchers under the supervision of a movement disorders specialist. Standardized diagnostic criteria and validated scales were used to minimize misclassification. Furthermore, patients with neuropsychiatric diseases that could confound the assessment of psychotic symptoms were excluded from the study.

### Study size

The study sample consisted of all eligible patients evaluated during the study period. No formal sample size calculation was performed, as this was an exploratory study primarily aimed at estimating prevalence and describing the clinical and sociodemographic profile of the cohort.

### Variables and statistical analysis

Quantitative variables were tested for normality using the Shapiro–Wilk test. Normally distributed variables were presented as mean ± standard deviation (SD), while non-normally ones were presented as median and interquartile range (IQR). Univariate comparisons between patients with and without PDP were performed using Student's *t* -test or Mann–Whitney U test for continuous variables, according to their distribution, and the chi-squared or Fisher's exact tests for categorical variables.

Given the exploratory nature of the study and the limited sample size, no formal correction for multiple comparisons was applied, and results should be interpreted as hypothesis-generating. A multivariable logistic regression analysis was conducted as an exploratory approach to assess potential independent associations with PDP. Variables were selected based on clinical relevance and univariate results, with the number of variables included in the model limited according to the number of outcome events to reduce the risk of overfitting and to ensure model stability.

Additionally, a sensitivity analysis was performed to estimate the prevalence of psychotic symptoms by including patients who did not fulfill the NINDS/NIMH criteria, either because they experienced only passage hallucinations, which are not included in these criteria, or because their symptoms did not meet the required frequency or duration. We also conducted a separate sensitivity analysis to estimate the prevalence of patients presenting exclusively with minor phenomena, irrespective of whether they met the criteria. A two-sided *p* -value < 0.05 was considered statistically significant. All analyses were performed using jamovi (The jamovi project), version 2.6.4.

### Ethical aspects

The study was approved by the Research Ethics Committee of Hospital Geral Roberto Santos (CAAE 56085222.1.0000.5028). All participants or their legal representatives provided written informed consent prior to inclusion in the study.

## RESULTS

### Participants

A total of 81 patients were screened for eligibility. Then, 6 patients were excluded: 3 due to comorbid bipolar disorder, 1 due to an inability to complete the assessment, and 2 who declined participation. Therefore, the final cohort comprised 75 patients, all of whom were included in the analysis. Among these, 72 (96.0%) were receiving levodopa therapy at the time of evaluation.

### Patient characteristics

The median age was 68 years (IQR: 59.5–74.5), and the mean age at onset was 56.0 ±  9.8 years. The median disease duration was 10 years (IQR: 6–14.5). 41 patients (54.7%) were male, and most self-identified as Mixed-race (48.6%) or Black (31.1%). According to the modified Hoehn & Yahr scale, 82.7% of patients had mild-to-moderate disease (stages 1–3), whereas 17.3% were classified as severe (stages 4–5).

### Prevalence and clinical features of PDP

We diagnosed PDP in 18 patients (24%) according to the NINDS/NIMH criteria ( Table 1 ). Minor phenomena were observed in 72.2% of patients with PDP, including false sense of presence (61.1%) and illusions (27.8%).

**Table 1:** Psychosis characteristics among patients with PD psychosis (n = 18)

Variable	n (%)
Minor phenomena	13 (72.2)
False sense of presence	11 (61.1)
Illusions	5 (27.8)
Hallucinations (any)	13 (72.2)
Visual	5 (27.8)
Auditory	2 (11.1)
Olfactory	1 (5.6)
Mixed (visual and auditory)	5 (27.8)
Delusions (any)	2 (11.1)
Preserved insight	16 (88.9)
MDS-UPDRS item 1.2	
Slight (score 1)	5 (27.8)
Mild (score 2)	10 (55.6)
Moderate (score 3)	1 (5.6)
Severe (score 4)	2 (11.1)

Abbreviations: MDS-UPDRS, Movement Disorders Society's Unified Parkinson's Disease Rating Scale; PD, Parkinson's disease.

Note: Results are presented as n (%) for categorical variables.

Passage hallucinations, which are not included in the NINDS/NIMH criteria, were analyzed separately. Hallucinations of any type were present in 72.2% of patients, with visual hallucinations occurring in 55.6%, often in association with other modalities. Delusions were less frequent, identified in 11.1% of patients with PDP.

Insight was preserved in most cases (88.9%). Regarding psychosis severity, as assessed by the MDS-UPDRS Part I, item 1.2, most patients had mild (55.6%) or slight (27.8%) symptoms.

### Factors associated with PDP

#### *Univariate analysis*

When comparing patients with and without PDP ( Tables 2 and 3 ), no significant difference was observed between groups regarding age (71.5 [61.3–74.5] vs. 68.0 [57.0–74.0], *p* = 0.483), age at onset (54.3 ±  8.3 vs. 56.5 ±  10.2, *p* = 0.407), disease severity according to the mH&Y ( *p* = 0.499), sex ( *p* = 0.648), self-reported race ( *p* = 0.248), or education ( *p* = 0.124). Patients with PDP had a longer disease duration (14.5 [11.0–17.0] vs. 9.0 [6.0–13.0] years, *p* = 0.009), lower MMSE scores (23.5 [19.3–27.0] vs. 27.0 [22.0–29.0], *p* = 0.041), a higher number of neuropsychiatric symptoms (4 [3.0–6.8] vs. 3 [2.0–4.0], *p* = 0.03) and greater NPI-Q severity scores (9 [6–14.8] vs. 6 [3–8.0], *p* = 0.018).

**Table 2:** Sociodemographic characteristics of patients with PD according to psychosis status

Variable	Non-PDP ( *n* = 57)	PDP ( *n* = 18)	*p* -value
Age, years	68.0 (57.0–74.0)	71.5 (61.3–74.5)	0.483
Age at onset, years	56.5 ± 10.2	54.3 ± 8.3	0.407
Male sex	32 (56.1)	9 (50.0)	0.648
Self-reported race			0.248
White	8 (14.0)	1 (5.6)	
Black	16 (28.1)	7 (38.9)	
Mixed-race	28 (49.1)	8 (44.4)	
Other	5 (8.8)	2 (11.1)	
Education, years	12 (5.0–12.0)	9 (5.0–12.0)	0.124

Abbreviations: IQR, interquartile range; PD, Parkinson's Disease; PDP, Parkinson's Disease Psychosis; SD, standard deviation.

Note: Results are presented as median [IQR] or mean ±  SD for continuous variables and n (%) for categorical variables.

**Table 3:** Clinical and treatment-related characteristics of patients with PD according to psychosis status

Variable	Non-PDP ( *n* = 57)	PDP ( *n* = 18)	*p* -value
Disease duration, years	9.0 [6.0–13.0]	14.5 [11.0–17.0]	0.009
Mild-to-moderate disease (mH&Y)	48 (84.2)	14 (77.8)	0.499
MMSE score	27.0 [22.0–29.0]	23.5 [19.3–27.0]	0.041
Cognitive impairment	29 (50.9)	12 (66.7)	0.241
Dementia	1 (1.8)	2 (11.1)	0.141
NPI-Q severity	6 [3.0–8.0]	9 [6.0–14.8]	0.018
NPI-Q distress	4 [1.0–8.0]	5.5 [3.0–13.0]	0.174
Number of neuropsychiatric symptoms (NPI-Q)	3 [2.0–4.0]	4 [3.0–6.8]	0.03
LEDD, mg/day	750 [600–1100]	1476 [942–1595]	0.003
Levodopa immediate release	54 (94.7)	17 (94.4)	1.000
Benserazide Carbidopa	50 (87.8) 4 (7.0)	17 (94.4) 0 (0.0)	
Levodopa controlled release	23 (40.3)	15 (83.3)	0.001
Levodopa dual release	2 (3.5)	0 (0.0)	1.000
Dispersible levodopa	5 (8.8)	4 (22.2)	0.205
COMT inhibitor	9 (15.8)	10 (55.6)	0.002
Dopamine agonist	19 (33.3)	10 (55.6)	0.091
MAO-B inhibitor	5 (8.8)	3 (16.7)	0.389
Amantadine	12 (21.1)	7 (38.9)	0.212
Atypical antipsychotic	4 (7.0)	3 (16.7)	0.348
Clozapine Quetiapine	2 (3.5) 2 (3.5)	1 (5.6) 2 (11.1)		
Antidepressant	20 (35.1)	5 (27.8)	0.566
Anticholinergic	4 (7.0)	2 (11.1)	0.626
Smoking history	15 (26.3)	11 (61.1)	0.007
Alcohol consumption history	19 (33.3)	10 (55.6)	0.091
Diabetes	10 (17.5)	6 (33.3)	0.191
Hypertension	24 (42.1)	8 (44.4)	0.861
Dyslipidemia	10 (17.5)	3 (16.7)	1.000
Depression	10 (17.5)	3 (16.7)	1.000
Previous stroke	0 (0.0)	3 (16.7)	0.012
Ischemic heart disease	2 (3.5)	1 (5.6)	0.567

Abbreviations: COMT, catechol-O-methyltransferase; IQR, interquartile range; LEDD, Levodopa equivalent daily dose; MAO-B, monoamine oxidase B inhibitor; mH&Y, modified Hoehn & Yahr scale, MMSE, Mini-Mental State Examination, NPI-Q, Neuropsychiatric Inventory Questionnaire; PDP, Parkinson's Disease Psychosis; SD, standard deviation.

Note: Results are presented as median [IQR] or mean ±  SD for continuous variables and n (%) for categorical variables.

Regarding treatment, patients with PDP had higher LEDD (1476 [942–1595] vs. 750 [600–1100], *p* = 0.003), more frequent use of controlled-release levodopa (83.3 vs. 40.3%, *p* = 0.001) and COMT-inhibitors (55.6 vs. 15.8%, *p* = 0.002). Additionally, smoking history (61.1 vs. 26.3%, *p* = 0.007) and previous stroke (16.7 vs. 0.0%, *p* = 0.012) were more frequent among patients with PDP. No significant differences were observed for other dopaminergic drugs or comorbidities.

#### *Multivariable analysis*

The exploratory multivariable logistic regression model, which included age, disease duration, LEDD, and MMSE score, is presented in Table 4 . In this analysis, no independent predictors of PDP were identified. Disease duration (OR: 1.06, 95% CI: 0.91–1.23, *p* = 0.442), age (OR: 0.99, 95% CI: 0.93–1.08, *p* = 0.988), and MMSE score (OR: 0.92, 95% CI: 0.80–1.05, *p* = 0.206) were not significantly associated with the presence of psychosis. Notably, higher LEDD showed a trend toward an association with PDP (OR: 1.001, 95% CI: 1.000–1.003, *p* = 0.076), although it did not reach statistical significance in this exploratory model.

**Table 4:** Multivariable logistic regression analysis including age, disease duration, LEDD, and MMSE

Variable	OR	95% CI	*p* -value
Disease duration	1.06	0.91–1.23	0.442
Age	0.99	0.93–1.08	0.988
LEDD ^a^	1.001	1.000–1.003	0.076
MMSE score	0.92	0.80–1.05	0.206

Abbreviations: CI, confidence interval; LEDD, levodopa equivalent daily dose; MMSE, Mini-Mental State Examination; OR, odds ratio.

Notes: Variables were selected based on clinical relevance and univariate analysis results. ^a^ For LEDD, three decimal places are shown to reflect the small increment per unit (mg).

### Sensitivity analysis

In the sensitivity analysis, the prevalence of any psychotic manifestation increased to 34.7% (n = 26) when including patients who did not meet the NINDS/NIMH criteria. Among the 8 additional patients identified, 6 presented with passage hallucinations alone or in combination with illusions and false sense of presence, while 2 exhibited illusions or false sense of presence. These patients did not satisfy the 1-month duration or frequency requirements, or they experienced frequent or continuous passage hallucinations, which are not considered in the NINDS/NIMH criteria. Furthermore, when analyzing the prevalence of minor phenomena alone (excluding delusions and formed hallucinations), irrespective of whether the diagnostic criteria for PDP were met, the rate was 30.7% (n = 23).

## DISCUSSION

The primary objective of this study was to estimate the prevalence of PDP in a Brazilian cohort. The demographic and clinical characteristics of our sample, including age, sex distribution, disease duration, and mH&Y staging were comparable to those reported in previous observational studies. 5 24 25 A distinctive feature of our cohort was its racial composition, with the majority of patients self-identifying as Mixed-race or Black.

Based on the NINDS/NIMH criteria, the prevalence of PDP was 24% ( *n* = 18). This result is consistent with the 23.6% reported by Mack et al., 24 and higher than the 13.9% found by Rodríguez et al., 26 yet lower than the rates observed by Fénelon et al. (60%), 25 Gordon et al. (50%), 12 and Stang et al. (38.4%). 6 Interestingly, our prevalence closely aligns with the 23.2% reported by Stang et al. at 10 years of disease duration.

It is worth noting that studies that did not strictly apply the NINDS/NIMH criteria have reported slightly higher prevalence rates, ranging from approximately 30 to 39%, 11 27 suggesting that these criteria may not capture the full spectrum of PDP. Importantly, the NINDS/NIMH criteria do not include passage hallucinations and require psychotic symptoms to be recurrent or frequent for at least 1 month.

In our cohort, eight additional patients presented with minor phenomena that did not fulfill these strict requirements. Among them, six reported passage hallucinations, although only three met the frequency or duration thresholds. If a broader diagnostic approach were applied, including minor phenomena irrespective of strict duration or frequency requirements, a greater number of cases would be identified. In our sample, this would correspond to 26 patients (34.7%) presenting any psychotic symptoms.

While the MDS-UPDRS Part I (item 1.2) incorporates minor phenomena as part of a continuum of severity, it remains somewhat restrictive due to its predetermined categories and may be limited in capturing the complexity and overlap of psychotic symptoms. A more comprehensive scale, incorporating frequency, duration, specific types of delusions or hallucinations, presence of insight, and impact on daily life, would likely provide a more accurate assessment of the PDP spectrum.

Visual hallucinations (VH) are commonly regarded as the hallmark of PDP, with a relatively homogeneous prevalence in literature, ranging from 22 to 38%. 28 While nonvisual hallucinations are not uncommon, they tend to occur in older patients and usually in association with VH. 1 29 Delusions are even less frequent, typically reported in 1 to 7% of cases. 1 In our sample, illusions and false sense of presence (minor phenomena considered by the NINDS/NIMH criteria) occurred with the same frequency as hallucinations (72.2%). The latter were predominantly visual (55.6%), a rate closely similar to that reported by Mack et al. 24 Insight was preserved in 88.9% of our PDP patients, though it is worth noting that its loss is associated with more severe psychosis, including delusions, multimodal hallucinations, poorer quality of life, and cognitive decline. 1

Minor phenomena have recently gained increasing attention, as they often emerge early in the course of the disease, sometimes even preceding motor manifestations and occurring in drug-naïve patients. 30 Reported prevalence varies widely, from 20 to 45% across studies, largely due to methodological differences and the fact that these symptoms have often been excluded from PDP definitions. The first systematic study including minor phenomena reported a prevalence of 25.5% out of 216 patients with PD, 31 while a later study by the same group reported a prevalence of 45%. 25 In a sensitivity analysis including patients presenting with any minor phenomena, irrespective of whether they met the NINDS/NIMH criteria, the prevalence in our cohort was 30.7%, a rate comparable to the 32.9% reported by Wang et al. 32

In our exploratory secondary analysis of clinical and treatment-related factors associated with PDP, univariate analysis suggested potential associations between PDP and longer disease duration, lower MMSE scores, a greater burden of neuropsychiatric symptoms (assessed by the NPI-Q), and higher LEDD, particularly with more frequent use of controlled-release levodopa and COMT inhibitors. These findings are partly consistent with previous studies. 11 24 26 33

Multiple studies have demonstrated an association between PDP and cognitive impairment. 34 35 36 Additionally, PDP has been linked to other neuropsychiatric symptoms 37 and a higher overall burden of non-motor symptoms. 33 Marsh et al. reported that 56% patients with PDP had at least one other comorbid psychiatric disturbance. 38

It is worth noting that the MMSE is not the preferred tool for cognitive screening in PD, as it has limited sensitivity for executive and visuospatial dysfunction, domains that are particularly relevant in PD, 22 which may have influenced the associations found here. However, it was selected due to its practicality, shorter administration time, and widespread use in clinical practice in Brazil. Furthermore, although the Montreal Cognitive Assessment (MoCA) is often preferred for its better sensitivity, it presents significant limitations in populations with lower formal education, such as ours. 39 Smoking history and previous stroke were also more frequent among patients with PDP, although these findings should be interpreted cautiously and considered exploratory. Drug-specific associations should also be interpreted with caution, as they likely reflect clinical management in advanced disease stages, rather than a direct causal contribution to psychosis.

We conducted an exploratory multivariable logistic regression model including age, disease duration, LEDD, and MMSE. Only a limited number of variables were included to ensure model stability. None of the predictors reached independent statistical significance, although LEDD showed a trend toward significance (OR: 1.001; 95% CI: 1.000–1.003; *p* = 0.076). The relationship between dopaminergic therapy and psychosis is complex, with levodopa often being considered a facilitating factor in the development of PDP. 7

We hypothesize that the total dopaminergic burden, rather than specific drug classes, may be more relevant to psychosis risk. Supporting this interpretation, Forsaa et al. 11 observed a dose-dependent relationship, with a 26% increased risk of incident PDP per 100 mg increase in baseline levodopa dose over 12 years of follow-up. Importantly, as Mack et al. 24 noted, current LEDD may not capture historical prescribing behavior, since treatment regimens are often adjusted in response to emerging psychotic symptoms.

This study has some important limitations. Its cross-sectional design and the small number of outcome events limit statistical power and preclude causal inference. Therefore, the results should be interpreted as exploratory and hypothesis-generating, especially regarding the associations found. The relatively small sample size, drawn from a convenience cohort at a tertiary outpatient clinic, may limit generalizability and introduce the possibility of selection bias. Patients with mild, uncomplicated presentation, who may have a lower risk of psychosis, and institutionalized patients with advanced disease, who may have a higher risk, are likely underrepresented. Furthermore, the high number of tests performed without correction for multiple comparisons increases the risk of type I error.

Additionally, PDP was assessed according to the NINDS/NIMH criteria, yet there is still considerable heterogeneity in how PDP is defined and measured across studies, which limits comparisons. 40 41 The use of the MMSE as a cognitive screening tool also represents a limitation, given its lower sensitivity in PD.

Despite these limitations, a major strength of this study is that it provides original data from a racially diverse Brazilian cohort, a population significantly underrepresented in the PDP literature. The study also contributes to the growing body of evidence on minor phenomena, which are increasingly recognized as early markers of PDP. Additionally, the study followed STROBE recommendations and used standardized instruments for cognitive and motor assessment, allowing for better comparison with international cohorts.

In conclusion, our findings are in line with the existing literature, demonstrating a 24% prevalence of PDP in a racially mixed Brazilian cohort using diagnostic criteria widely applied in other studies. Notably, the racial profile did not appear to modify the prevalence or clinical expression of PDP when compared with the predominantly White cohorts reported in prior studies. The findings from the exploratory secondary analysis of associated factors were also mostly consistent with previous reports, although they should be interpreted as hypothesis-generating. Psychotic symptoms remain underrecognized and often neglected within the broader spectrum of PD manifestations, yet our results reinforce that they are far from uncommon and should not be overlooked in clinical practice. Finally, we highlight the need for a standardized, multilingual instrument to diagnose, grade, and monitor PDP severity, as the current NINDS/NIMH criteria do not fully capture the heterogeneity of its presentation and phenomenology.
